# Soil Amendments with Spearmint, Peppermint and Rosemary Enhance the Community of Free-Living Nematodes and Improve Soil Quality, While Having Strikingly Different Effects on Plant Growth

**DOI:** 10.3390/life12081121

**Published:** 2022-07-26

**Authors:** Maria D. Argyropoulou, Maria Karmezi, Maria Tsiafouli, Dimitris Chalkos, Areti Bountla, Despoina Vokou

**Affiliations:** 1 Department of Zoology, School of Biology, Faculty of Sciences, Aristotle University, 54124 Thessaloniki, Greece; mkarmezi@bio.auth.gr; 2 Department of Ecology, School of Biology, Faculty of Sciences, Aristotle University, 54124 Thessaloniki, Greece; tsiafoul@bio.auth.gr (M.T.); chalkosd@gmail.com (D.C.); vokou@bio.auth.gr (D.V.); 3 Hellenic Agricultural Organization-DEMETER, Soil and Water Resources Institute, 57001 Thermi, Greece; a.bountla@swri.gr

**Keywords:** aromatic plants, *Mentha spicata*, *Mentha piperita*, *Rosmarinus officinalis*, free-living nematodes, soil food web, feeding groups, nematode indices, metabolic footprint, essential oils

## Abstract

Sustainable farming practices aim to replace agrochemicals with plant-based alternatives to increase productivity and soil quality. To evaluate the potential use of aromatic plants as soil amendments in tomato seedbeds, in a greenhouse experiment, we used spearmint, peppermint, and rosemary, separately, as soil amendments, in pots sown with tomato, and studied their effect on seedling growth, soil nutrients, and the soil nematode community in terms of trophic and functional structure, metabolic footprint, and genera composition. Non-amended soil was used in the control pots. We further explored the dynamics of the plant–soil–nematode interactions by using aromatic plants at different stages of decomposition (0, 28, and 56 days). Incorporating aromatic plants into the soil led to the proliferation of free-living nematodes, especially of the opportunistic kind, resulting in vigorous and enriched soil. This was more pronounced in the case of the spearmint and peppermint, which also increased the tomato growth. The high soil nutritional status and enhanced plant growth were most prominent when the aromatic plants were left for 28 days to decompose in the soil before sowing. Compared with the mint plants, the rosemary had similar, yet less intense, effects on the soil community, but completely inhibited the growth of the tomato seedlings. Therefore, it is not recommended for use as a soil amendment in tomato seedbeds.

## 1. Introduction

In sustainable agriculture and organic farming, agricultural practices aim to reduce agrochemicals and contribute to the preservation of soils, water, and biodiversity. In this context, the research on plant-based alternatives to chemical fertilizers, herbicides or pesticides is gaining in popularity. In the Mediterranean area, the incorporation of plant residues into the soil has been used since antiquity as a method to improve productivity. So far, a plethora of plant materials have been tested as potential soil amendments. Among them, aromatic plants, which are abundant in this area, are important candidates because of their essential oils, which affect plant–plant and plant–microbe interactions [1,2,3]. These plants have been shown to enhance soil metabolism and microbial activity, to suppress weeds, and to inhibit plant pathogens [4,5,6].

The work presented here is part of a wider project aiming to assess the performance of aromatic plants as soil amendments in tomato seedbeds. Tomato was chosen as a case-study because it is among the ten most important crops in South Eastern Europe [7]. We focused on seedbed management, because aromatic plants are costly materials and their use as soil amendments might be advisable only in small-scale applications [5]. More specifically, the aboveground parts of spearmint (*Mentha spicata* L.), peppermint (*Mentha piperita* L.), and rosemary (*Rosmarinus officinalis* L.) were incorporated into the soil without prior composting, and various plant-growth and soil-dynamic parameters were assessed. Using the same setting as in this study, Karamanoli et al. [8] previously studied the quantitative and qualitative changes that essential oils undergo as the parts of spearmint, peppermint and rosemary decay in the soil, whereas Ainalidou et al. [9] examined how the soil microbial community changes with time, as these three aromatic plants decay, and how they affect tomato growth, photosynthesis, and metabolism. Accordingly, as a final step in this multidimensional research examining the potential use of aromatic plants as soil amendments, we focus on the community of soil nematodes in this study.

Many studies have been conducted on the various properties of spearmint, peppermint, and rosemary that are of interest to sustainable farming. For example, Chalkos et al. [10] showed that spearmint compost, when added at a rate of 4% to 8%, stimulated tomato growth, increased soil bacterial and fungal abundance, and inhibited weed emergence. On the other hand, spearmint essential oil and/or its constituents were found to inhibit seedling emergence and the growth of tomato, cotton, and several weeds [11], whereas the major essential-oil constituents of these three aromatic plants, including carvone, menthol, cineol, and camphor, were found to strongly inhibit both lettuce germination and seedling growth in laboratory experiments [12,13]. The essential oils of all three plants were also proven to be efficient against phytoparasitic nematodes [14,15], while improving through their root exudates the growth of infested sunflower plants [16]. The main reason for the often contrasting results stems from differences between experimental settings, as well as from the different forms of the plant materials used by researchers, e.g., plant macerates, composts, essential oils, and specific essential oil constituents. Indeed, Ntalli et al. [17] suggested that less-refined botanical preparations possess milder but more diverse biochemical modes of action, while Vokou et al. [12] showed that the activity of an essential oil compound may be altered due to its antagonistic or synergistic interactions with other constituents.

Due to the multifaceted biological activity of aromatic plants and their constituents, the evaluation of their potential use as soil amendments should consider their effects on both plant growth and soil communities that are vital for soil functionality, such as microbes and soil nematodes. Soil bacteria and fungi, as well as the free-living nematodes that graze on them, are the main actors in important soil processes, such as the decomposition of organic residues, nitrogen mineralization, nutrient cycling, and the formation of humic substances [18,19,20]. Since nematodes occur at multiple levels of the soil food web, the study of their community, in terms of both trophic types and life strategies, offers a better insight into the functionality of the food web and, hence, the nutritional status of the soil [19,21]. However, we should note that, apart from assessing the efficacy of botanicals against root-knot nematodes and a few other plant parasites, there are few studies on their effects on free-living soil nematodes [17,22,23,24,25].

Here, we assess and compare the effects of the three aromatic plant amendments (i.e., spearmint, peppermint, and rosemary) on the soil nematode community, by analyzing its trophic and functional structure, as well as its composition in terms of nematode genera. Furthermore, we investigate whether the stage of decomposition of the aromatic plants used can determine the soil community structure, the soil-nutrient status and the growth of the crop plant. The results of this study, together with those of Karamanoli et al. [8] and Ainalidou et al. [9], will allow us to evaluate the suitability of spearmint, peppermint, and rosemary as soil amendments and biostimulants. Most importantly, they will offer a deeper understanding of how aromatic plants and their essential oils affect soil dynamics.

## 2. Materials and Methods

### 2.1. Greenhouse Experiment

We conducted a greenhouse pot experiment at the farm of the Aristotle University of Thessaloniki, Greece. Dry material of three aromatic plants, all members of the family Lamiaceae, viz. spearmint (*Mentha spicata*), peppermint (*Mentha piperita*), and rosemary (*Rosmarinus officinalis*), was used as soil amendment. These plants grow in the wild in Greece and are also cultivated. The material used in the experiments was purchased from a commercial supplier. For spearmint and peppermint, given their herbaceous character, the whole aboveground biomass was used, but for rosemary, only the upper green part of shoots was used. All samples were cut into small pieces. The quantitative and qualitative features of the essential oils of the three aromatic plants were studied previously [8]; the yield and main constituents of the three essential oils are presented in Table 1.

The soil used for the experiments was from the farm of the Aristotle University of Thessaloniki, from a field left to lie fallow for at least 10 years. It consisted of 32% clay, 56% silt, 12% sand, 3.1% organic matter, and 1.7% CaCO_3_, with Corg/Norg equal to 6.8 (estimations by Kadoglidou et al. [5]).

The aromatic plants were mixed separately with the soil at a rate of 4%, after the soil passed through a 10-mm-pore sieve. Each soil mixture made with one of the three aromatic plants was placed in a 2-kg pot inside a greenhouse. Pots were initially watered to full capacity and then every other day. Tomato seeds var. EZ Noam were sown at three time points; one set of pots was sown at the start of the experiment (SOE), i.e., at day 0, a second set was sown 28 days after SOE, and a third set was sown 56 days after SOE. Ten tomato seeds were sown per pot each time. In this way, the aromatic plants mixed with soil were at three different decomposition stages at the time of sowing (0 D, 28 D, and 56 D). Non-amended soil was also used as a control treatment. Four weeks after sowing, the plants were uprooted for plant-growth measurements, estimation of soil properties, and nematode extraction. Thus, in all treatments, the age of tomato seedlings was the same (up to 28 days). To examine the effect that the crop plant might have on soil communities and properties, all treatments were doubled in pots that were not sown. As a result, in the whole experiment, there were four types of amendments (Ms: spearmint, Mp: peppermint, Rs: rosemary, and C: control), three stages of aromatic plant decomposition (0, 28, and 56 days after incorporation into the soil), and two types of pot (with or without tomatoes sown). In total, there were 24 treatments (4 × 3 × 2), and for each treatment there were three replicate pots. The temperature range over the whole duration of the experiment was 15–24 °C and the relative humidity was 45–60%.

### 2.2. Sampling and Plant and Soil Analyses

We conducted our samplings 28 days after seeds were sown. We should note that since the aromatic plants used as soil amendments continued to decompose, even after sowing, the 0, 28, and 56 D of amendment decomposition at the time of tomato sowing corresponded to 28, 56, and 84 days of amendment decomposition, respectively, at the time of sampling. Tomato seedlings were gently uprooted and weighed, after soil particles were removed from the roots.

From each pot, we took a subsample of 200 cm^3^ for soil analyses. We estimated organic carbon and nitrogen by the method developed by Kjeldahl according to Allen [26], extractable phosphorus according to Olsen [27], and inorganic N (ammonium and nitrate ions) using the distillation method [28], using the reagents MgO and “Devarda’s alloy” (mixture of Cu, Al, Zn), for the determination of ammonium ions and nitrates, respectively. We also estimated the interchangeable potassium (K), and magnesium (Mg) cations. For this, we treated soil samples with ammonium acetate and filtered them. We measured K concentrations by using a flame photometer, while for Mg concentrations, we used atomic absorption spectrophotometer.

### 2.3. Nematode Extraction and Identification

From each pot, we took a soil sample of 150 cm^3^. We extracted nematodes by using Cobb’s sieving and decanting method, as proposed by s’Jacob and Van Benzooijen [29]. After counting the living nematodes under the stereomicroscope, we heat-killed and fixed them with 4% formaldehyde. From each sample, we identified under the microscope 100 randomly selected nematodes to genus level, using Bongers’ identification key [30]. Genera were assigned to trophic groups according to Yeates et al. [31] and classified along the colonizer–persister gradient (c-p values) of life strategies according to Bongers [32] and Bongers and Bongers [33].

### 2.4. Nematode Functional Indices

For the calculation of indices, we used the automated calculation system for nematode-based biological monitoring (NINJA) [34]. The Maturity Index (MI) for free-living nematodes and the Plant Parasitic Index (PPI) for plant feeding nematodes, both of which indicate the successional status of the community, were calculated according to Bongers [32]. Calculations of the Enrichment index (EI), the Basal Index (BI), the Channel Index (CI), and the Structure Index (SI), which indicate the functional structure of the food web, were performed according to the weighted faunal analysis proposed by Ferris et al. [35]. More specifically, the EI indicates soil enrichment with organic material, mirroring the increases of enrichment opportunists, mainly bacterial feeding nematodes, which respond rapidly to increases in food. The CI indicates the degree of fungal participation in the decomposition pathway, the BI is an indicator of the pervasiveness of nematodes that are tolerant to stress factors, e.g., limitation of resources and/or adverse environmental conditions, while the SI is an indicator of nematodes with high longevity, body size, and disruption sensitivity. The metabolic footprint (MF) was calculated according to Ferris [36]; this is an index of carbon utilization by nematodes and corresponds to the sum of the lifetime amount of carbon gained, partitioned into growth, egg production, and respiration. The MF is expressed in standardized carbon units per gram of dry soil.

### 2.5. Statistical Analysis

In order to evaluate the effects of soil amendment (Ms, Mp, Rs, C), stage of decomposition of the aromatic plants applied (0 D, 28 D and 56 D), and tomato presence (T: with tomato, NT: without tomato) on soil nutrients and nematodes, we used permutation analyses of variance (PERMANOVA; [37]) for each response variable. The same analysis was used in the case of plant growth (evidently, only with data from sown pots). We should note that data regarding plant growth at 0 D and 28 D are from Ainalidou et al. [9].

All PERMANOVA analyses were performed with “amendment” (Ms, Mp, Rs, C) as fixed factor, “decomposition stage of aromatic plants” (0 D, 28 D, 56 D) nested within “amendment”, and “tomato presence” (T, NT) nested within the factors “decomposition” and “amendment”. For all cases, we used 4999 permutations; deviance of dissimilarities was the distance measure to generate dissimilarity matrices for the data. Pairwise a posteriori tests were performed among levels of the following factors: (a) “amendment”, (b) “decomposition” within factor “amendment” and (c) “tomato presence” within factor “decomposition” within factor “amendment”. For these analyses, we used the Fortran software, PERMANOVA [37].

In order to compare the effect of amendment and decomposition on the generic structures of nematode communities, all samples and nematode-genera abundances were ordinated by means of Canonical Correspondence Analysis (CCA) in PAST v3.2 [38].

## 3. Results

**Figure 1:**
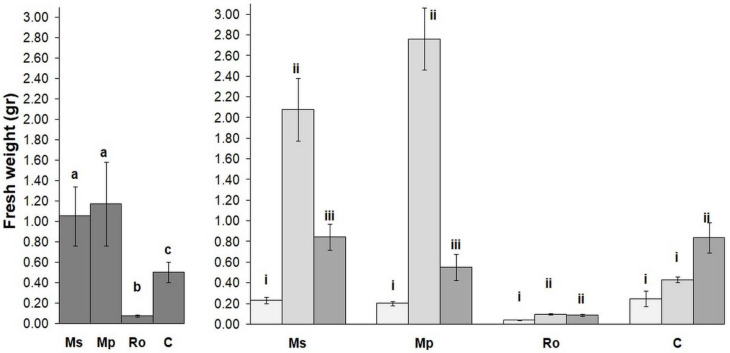
Fresh weight (g) of tomato plants (mean ± SE) for the four types of amendment (**left diagram**) and for the three stages of decomposition of the aromatic plants used as amendments (**right diagram**). Treatments were compared by means of PERMANOVA (Table S1). Superscripts over bars, a, b, and c, indicate significant differences between amendments (Ms: spearmint, Mp: peppermint, Ro: rosemary, C: control). Superscripts i, ii, and iii indicate significant differences between stages of amendment decomposition in soil (bars in light gray: 0 D, bars in middle gray: 28 D, bars in dark gray: 56 D). Data regarding 0 D and 28 D are from Ainalidou et al. [9].

**Figure 2:**
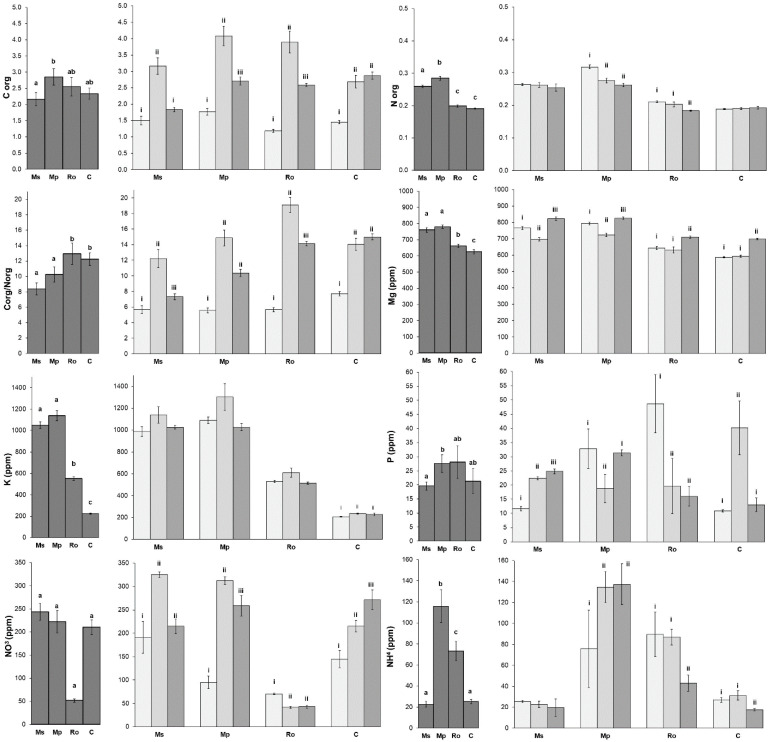
Soil parameters (mean ± SE) for the four types of amendment (**left diagrams**) and for the three stages of decomposition of the aromatic plants used as amendments (**right diagrams**). Treatments were compared by means of PERMANOVA (Table S2). Superscripts over bars, a, b, and c indicate significant differences between amendments (Ms: spearmint, Mp: peppermint, Ro: rosemary, C: control). Superscripts i, ii, and iii indicate significant differences between stages of amendment decomposition in soil (bars in light gray: 0 D, bars in middle gray: 28 D, bars in dark gray: 56 D).

**Figure 3:**
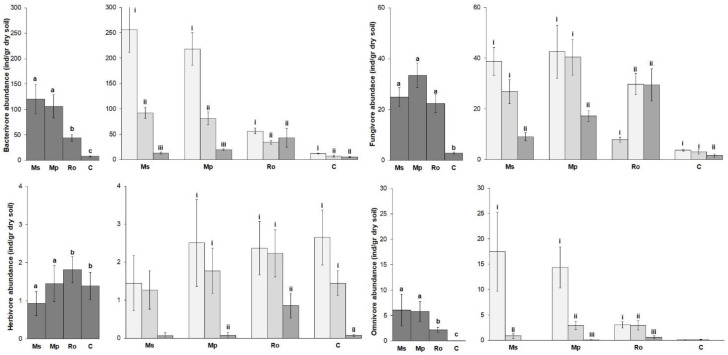
Abundance (mean ± SE) of the best-represented nematode trophic groups for the four types of amendment (**left diagrams**) and for the three stages of decomposition of the aromatic plants used as amendments (**right diagrams**). Treatments were compared by means of PERMANOVA (Table S3). Superscripts over bars, a, b, and c, indicate significant differences between amendments (Ms: spearmint, Mp: peppermint, Ro: rosemary, C: control). Superscripts i, ii, and iii indicate significant differences between stages of amendment decomposition in soil (bars in light gray: 0 D, bars in middle gray: 28 D, bars in dark gray: 56 D).

**Figure 4:**
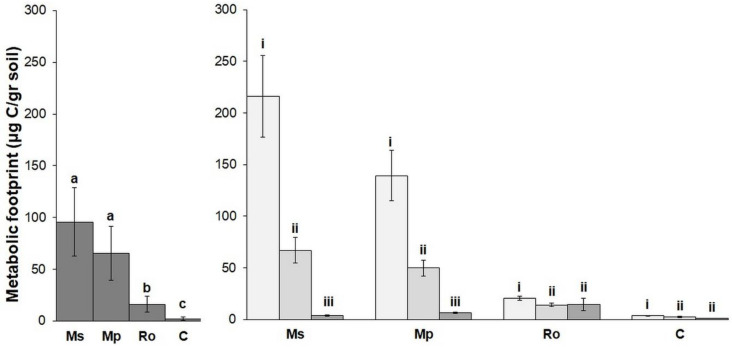
Metabolic footprint expressed as carbon units per g dry soil (mean ± SE) for the four types of amendment (**left diagram**) and for the three stages of decomposition of the aromatic plants used as amendments (**right diagram**). Treatments were compared by means of PERMANOVA (Table S3). Superscripts over bars, a, b, and c, indicate significant differences between amendments (Ms: spearmint, Mp: peppermint, Ro: rosemary, C: control). Superscripts i, ii, and iii indicate significant differences between stages of amendment decomposition in soil (bars in light gray: 0 D, bars in middle gray: 28 D, bars in dark gray: 56 D).

**Figure 5:**
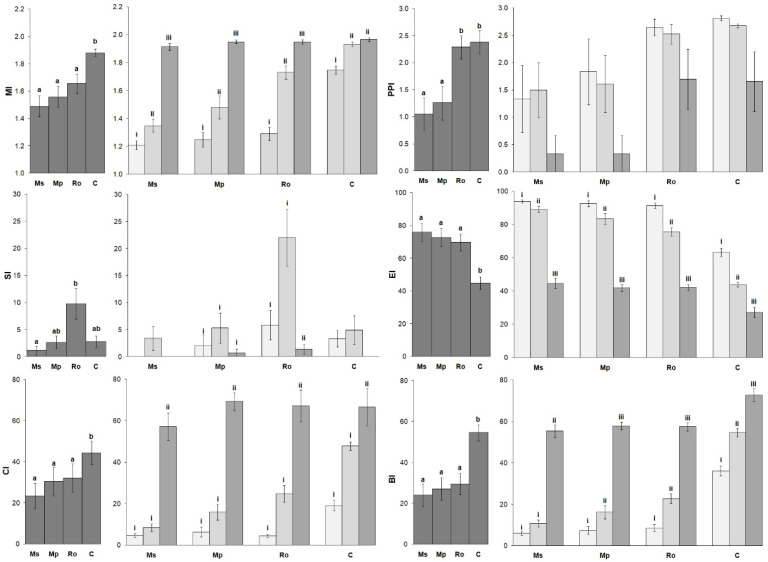
Values (mean ± SE) of the nematode functional indices for the four types of amendment (**left diagrams**) and for the three stages of decomposition of the aromatic plants used as amendments (**right diagrams**). Treatments were compared by means of PERMANOVA (Table S3). Superscripts over bars, a and b, indicate significant differences between amendments (Ms: spearmint, Mp: peppermint, Ro: rosemary, C: control). Superscripts i, ii, and iii indicate significant differences between stages of amendment decomposition in soil (bars in light gray: 0 D, bars in middle gray: 28 D, bars in dark gray: 56 D).

The results of the PERMANOVA regarding the effect of the aromatic plants used as soil amendments (Ms, Mp, Ro, and C) and of their decomposition stage (0 D, 28 D, and 56 D) on the growth of the tomato seedlings are shown in Table S1, while the PERMANOVA results regarding the effects of the two previous factors together with that of the presence of tomato on soil nutrients, nematode trophic groups, and nematode functional indices are shown in Tables S2 and S3. According to these analyses, both the type of amendment and the decomposition stage of the aromatic plants significantly affected all the individual variables studied, whereas the presence of tomato had no effect on seventeen out of the twenty studied variables. For each individual variable, in Figure 1, Figure 2, Figure 3, Figure 4 and Figure 5, we present the overall effects of the amendments (left diagrams), as well as the changes due to the aromatic plant decomposition stage within each type of amendment (right diagrams). We should note that since the presence of tomato had no influence on the soil and nematode variables, in Figure 2, Figure 3, Figure 4 and Figure 5, the data from the pots with and without tomato are pooled together.

The mean fresh weight of the plant seedlings for all the amendments is given in Figure 1 (left diagram). Compared to the control (C), the weight of the seedlings in the spearmint (Ms) and peppermint (Mp) treatments was significantly higher, whereas it was far lower in the rosemary (Ro) treatment. In fact, after their emergence, the tomato seedlings in the rosemary treatment did not grow at all. For the Ms and Mp treatments (Figure 1, right diagram), the fresh tomato weight was highest when the seeds were sown 28 days after the start of the decomposition process (28 D). At 56 D, the fresh-weight values of the two mint treatments were higher compared to those at 0 D.

Most of the soil nutrients exhibited higher values in the peppermint (Mp) and spearmint (Ms) treatments (Figure 2), but the Corg/Norg values displayed the opposite pattern, since they were higher in the rosemary (Ro) and control (C) treatments. In most cases, the Ro treatment was more similar to the non-amended control (C) than it was to the two mint treatments. Regarding the effect of the aromatic plant decomposition in the amended soils, Corg, the ratios of Corg/Norg, K, and NO_3_ were highest at 28 D, while Mg was highest at 56 D. Only Norg exhibited its highest values at 0 D. The changes in P and NH_4_ during the decomposition process of the aromatic plants did not follow any specific pattern.

The mean values of the nematode trophic group abundances are shown in Figure 3. Bacterivores overdominated the nematode community in all the treatments. Their abundance, together with that of the fungivores and omnivores (Figure 3, left diagrams), was highest in the spearmint and peppermint treatments (Ms, Mp), lower in the rosemary (Ro) treatment, and even lower in the control (C). On the other hand, the abundance of herbivores was higher in the (Ro) treatment and in the control. Regarding the effect of the stage of decomposition on the nematode community (Figure 3, right diagrams), the abundance of all the trophic groups was highest at 0 D, lower at 28 D, and even lower at 56 D. In all these cases, the trend was more pronounced in the spearmint and peppermint treatments (Ms, Mp). In the rosemary (Ro) treatment, the above-mentioned trend was not straightforward. For example, there was no significant difference in the abundance of bacterial feeders between 28 D and 56 D, while in the case of the fungal feeders, the abundance was lowest at 0 D.

The mean values of the nematode metabolic footprint and the nematode functional indices for the different treatments are shown in Figure 4 and Figure 5. The values of the nematode metabolic footprint (Figure 4) followed the same trend as the abundance of free-living nematodes; they were highest in the Ms and Mp treatments and lower in the Ro and the control (C). The same trend was also observed for the EI (Figure 5), although in this case, the differences between the amended soils were not significant. The values of PPI, on the other hand, followed the herbivore trend; they were higher in the Ro and C. The MI, BI, and CI indices were equally low in all the amended soils and high in the control. Regarding the effect of the stage of decomposition on the nematode indices, the metabolic footprint (Figure 4) and the EI and PPI values (Figure 5) were highest at 0 D, lower at 28 D, and even lower at 56 D. The MI, CI, and BI indices increased with longer durations of aromatic plant decomposition, while the SI was highest in the 28 D pots. In general, most of the nematode indices distinguished botanical treatments from the control and were significantly affected by the stage of aromatic plant decomposition.

**Figure 6:**
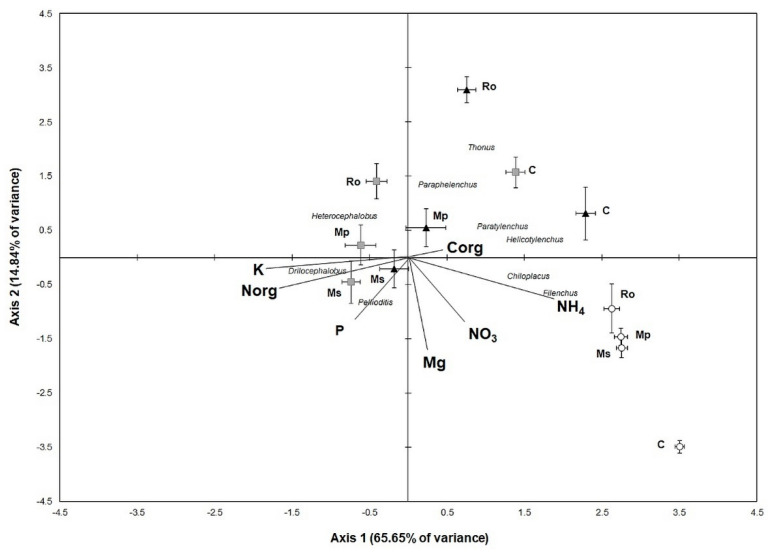
Canonical Correspondence Analysis (CCA) ordination diagram with soil samples, nematode genera, and soil variables (arrows). Each point represents mean values of sample scores (±SE) for each treatment (Ms: spearmint, Mp: peppermint, Ro: rosemary, C: control, squares: 0 D, triangles: 28 D, circles: 56 D). Only genera with high positive and negative scores are depicted.

The soil samples, nematode genera, and soil variables were ordinated along the two axes of the Canonical Correspondence Analysis (Figure 6). On the left part of the diagram, the 0 D-amended soil samples are ordinated along with K, N, and P, along with the genera, *Pelioditis* and *Drilocephalobus*. On the right lower part of the diagram, all the 56 D samples are ordinated together with NH_4_, NO_3_, and the genera, *Chiloplacus* and *Filenchus*. Moreover, all the control samples are ordinated on the right semi-plane. The most pronounced changes in the genera structure of the nematode community seemed to have occurred between 28 D and 56 D in the Ms and Mp treatments, as indicated by the sample distances along Axis 1, which reflects most of the data variance (65.65%). Regarding the differences between the aromatic plant amendments, the most pronounced was between the Ro and the Ms and Mp treatments at 28 D, which is reflected mostly along Axis 2. The fact that the second axis explains less than 15% of the data variance indicates that the effect of the stage of amendment decomposition was much stronger than the effect of the amendment per se on the alterations in the genera composition of the nematode community.

**Figure 7:**
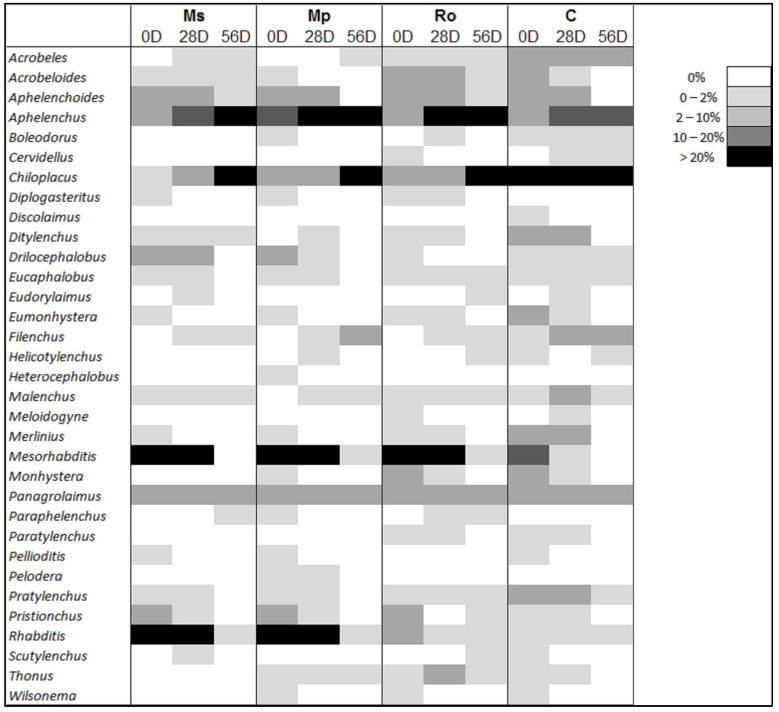
Percentage contribution of nematode genera to the total nematode community for each combination of amendment type (Ms: spearmint, Mp: peppermint., Ro: rosemary, C: control) and stage of aromatic plant decomposition in soil (0 D, 28 D, and 56 D).

Figure 7 shows the percentage contribution of the nematode genera to the total community for each treatment. *Mesorhabditis* dominated at the start of the experiment (0 D) in all the amended soils, while *Rhabditis* dominated only in the Ms and Mp treatments; the lowest contribution of both genera to the community of the amended soils was detected at 56 D. The opposite was observed for *Aphelenchus* and *Chiloplacus*: their contribution was high at 56 D and at 28 D. *Chiloplacus* and, to a lesser extent, *Aphelenchus* were the most abundant genera in the control samples and in the 56 D samples of the amended soils. *Diplogasteritus, Heterocephalobus, Paraphelenchus*, and *Pelodera* were not detected in the control samples; all four taxa made consistently low contributions in the amended soils. *Discolaimus* was detected in the control but not in the amended samples, and *Panagrolaimus* was the only genus that was equally present in all.

## 4. Discussion

In a greenhouse experiment, we used three aromatic plants, namely spearmint (Ms), peppermint (Mp), and rosemary (Ro), separately, as soil amendments in pots sown with tomato and studied their effect on (i) the tomato growth, (ii) the soil nutrients, and (iii) the soil nematode community. By using aromatic plants at different stages of decomposition (0 D, 28 D, and 56 D), we also evaluated the effect of the stage of the amendment decomposition. To examine the effect of the crop plant on (ii) and (iii), all the treatments were doubled, but without sowing tomato.

### 4.1. Presence of the Crop Plant

Plants are the main drivers of changes in the soil ecosystem. They provide food not only to root herbivores, such as plant-feeding soil nematodes, but also to microbivores, such as bacterivorous and fungivorous nematodes, by forming litter above and belowground and by releasing organic material from their roots as exudates [39,40]. However, in our experiment, the presence of tomato did not influence either the soil nutrients or the characteristics of the soil nematode community. The most probable explanation is the young age of the tomato seedlings. The root system of seedlings up to 28 days old was small and was, therefore, unable to enhance either the grazing or the detritus soil food web.

### 4.2. Spearmint, Peppermint, and Rosemary as Soil Amendments

The spearmint and peppermint (Ms, Mp) amendments resulted in heavier tomato seedlings and in higher concentrations of soil nutrients, i.e., organic nitrogen, magnesium, potassium, and phosphorus, evidently as a result of the nutrient release from the organic material added to the soil. Their effects on the soil nematodes were not uniform; the free-living nematodes proliferated significantly, especially the group of bacterivores, followed by that of fungivores, but the herbivores exhibited the opposite response. The essential oils of both *M. spicata* and *M. piperita* are considered to have nematicidal properties, at least when tested against crop-damaging plant-parasitic nematodes [14,15]. Contrasting effects on free-living and herbivorous nematodes have also been reported for aromatic plant amendments (e.g., anise, parsley, and rucola [24,25]) and other plant-based nematicides (e.g., *Melia azedarach* preparations [17]). It seems that apart from potential nematicidal activity, botanicals offer a significant trade-off to microbivorous nematodes by providing food to soil microflora. Indeed, Ainalidou et al. [9] found that spearmint and peppermint, when mixed with soil, enhanced almost all soil microbial groups. Εvidently, this subsequently enhanced the bacterivorous, fungivorous and omnivorous nematodes through the soil food chain. These changes are reflected in the high values of the metabolic footprint and the Enrichment Index in the Ms and Mp treatments, indicating vigorous and enriched soil, respectively, as well as in the low values of the Channel Index, which indicate an enhanced bacterial decomposition pathway. The low values of the Maturity Index in the Ms and Mp treatments also indicate pre-mature and more productive soil [41]. Indeed, when microbivorous nematodes feed on microflora, they excrete nutrients that are in excess of their metabolic needs in mineral or readily mineralizable forms, increasing soil quality [42,43]. Thus, both the mint amendments improved the nutritional status of the soil and enhanced the plant growth through a bottom-up effect on the soil community.

The effects of spearmint and peppermint on the plant, soil, and nematode variables that we examined, in most cases, did not differ significantly from each other, most probably because of the structural and chemical similarities of the two aromatic plants. Both mint plants are herbaceous, with soft leaves that degrade rapidly [8], and contain similar amounts of essential oils, which do not differ greatly from each other qualitatively; in the essential oils of both plants, menthol and isomenthone are the major constituents (Table 1). On the other hand, rosemary (Ro) is an evergreen shrub with hard, slowly degrading leaves that contain essential oils, which are rich in such plant-growth-inhibiting allelochemicals as cineol, camphor, and a-pinene [12,13], and persist for long durations in the soil environment [8]. As a result, the Ro amendment had a very strong negative impact on the growth of the tomato seedlings, contrary to the Ms and Mp. According to Ainalidou et al. [9], decaying rosemary leaves block the central metabolic pathways in tomato seedlings, whereas their effects on soil microflora are similar to those of decaying spearmint and peppermint macerates, except they are less pronounced. The latter probably dictated the responses of the microbivorous nematodes and omnivores to the Ro amendment, which were similar to, yet less pronounced than, the responses to the Ms and Mp. These mild changes in both microbes and microbivorous nematodes in response to the Ro amendment were probably due to the high lignin content of rosemary leaves, which makes their decomposition more difficult compared to the two herbaceous mint plants, the softer tissues of which provide a more labile food source to microbia, as indicated by the low Corg/Norg values from the Ms and Mp.

### 4.3. Decomposition Stage of Aromatic Plants

The stage of decomposition of the aromatic plants significantly modified their performance as soil amendments. In general, in 0 D treatments, the abundance of nematode trophic groups and their metabolic footprint were the highest, gradually decreasing with increasing levels of decomposition of the plant material added (28 D, 56 D). The pattern of a rapid initial increase, followed by a decrease, as the decomposition of the aromatic plants advanced was most clearly recorded in the Ms and Mp treatments, mirroring precisely the changes in the microbial biomass recorded by Ainalidou et al. [9], who used the same experimental setup. Ιn the Ro treatments, the response of the free-living nematode groups to the organic input was less pronounced and, in the case of the fungivores, it was delayed, with their highest abundance recorded at 28 D. Apart from the differences in the decomposition rates of the three aromatic plants, Karamanoli et al. [8] found that the essential-oil content of spearmint and peppermint soil mixtures decreased by approximately 90% after 30 days, while in a rosemary mixture, it decreased much more slowly. In our experiment, at 56 D, the trophic group abundances and metabolic activity were higher in the Ro than in the Ms and Mp treatments. This indicates the faster depletion of organic resources in the Ms and Mp treatments compared to the Ro. However, comparisons of all three botanical treatments with the control at 56 D showed that at the time of our final sampling, i.e., 84 days (56 + 28) after the incorporation of the aromatic plants in the soil, their effect on the abundance and metabolism of the free-living nematodes was still evident.

The responses of the herbivorous nematodes to the amendments differed from those of the microbivorous trophic groups. Although a gradual decline in their numbers was observed over the course of their decomposition, there was not an initial increase, as was the case with the microbivores. In fact, at 0 D, the herbivore abundance was highest in the control treatment, decreasing gradually with time, without the incorporation of any decaying aromatic plants. We should note that the abundance of herbivores at the beginning of our experiment was very low. This was partly due to our experimental setup; the sieving of the soil before the incorporation of the aromatic plants removed root fragments and, probably, the nematodes associated with them. Even if some of them had survived this procedure, the roots of the tomato seedlings would have been too small to support large populations of plant-feeding nematodes. Thus, the most probable reason for the gradual decline in the herbivores in all the pots was the absence of food.

The decline in the nematode abundance and metabolic activity due to the decomposition of aromatic plants added to the soil was accompanied by dramatic changes in the composition of the nematode genera, which were even greater than the changes caused by the different amendments (Figure 6). The nematodes that were favored most by the organic inputs were the cp-1 bacterivores, mainly *Rhabditis* and *Mesorhabditis*, which dominated at 0 D and 28 D, but reduced at 56 D (Figure 7). These genera have short life cycles and high reproductive potential; they mirror more closely than other nematodes the bloom of bacteria and are, therefore, described as enrichment opportunists [21]. The modifications of the Enrichment Index (EI) with the decomposition of the aromatic plants mirrored the rapid proliferation and subsequent reduction of these opportunistic genera. At later stages of decomposition, the enrichment opportunists were gradually replaced by general opportunists with longer life cycles, such as the cp-2 bacterivorous *Chiloplacus* and the cp-2 fungivorous *Aphelenchus*, leading to higher values on the Maturity Index, the Channel Index, and the Basal Index at 28 D and, in particular, at 56 D.

The majority of the soil nutrients displayed their highest values at 28 D and 56 D. The same held for the growth rate of the crop plant. More specifically, our heaviest tomato seedlings were found at 28 D. It seems that while the incorporation of aromatic plants into the soil enhanced the microbes and microbivorous nematodes within the first 28 days (treatment 0 D), it took another month for the microbivores to enrich the soil with nutrients, via their metabolic activity, and to promote plant growth (treatment 28 D). At later stages of aromatic plant decomposition (56 D), the results of the organic inputs were still evident, but far less pronounced.

## 5. Conclusions

Incorporating all three aromatic plants into the soil enhanced the free-living soil nematodes, resulting in vigorous and enriched soil, as indicated by the nematode functional indices. This was more pronounced in the case of the two mint herbs (Ms and Mp), which also promoted the growth of the tomato seedlings. The high soil nutritional status and enhanced plant growth were most prominent when the aromatic plants were left for 28 days to decompose in the soil before sowing. Compared with the spearmint and peppermint, the rosemary had similar, yet less intense, effects on the soil community but completely inhibited the growth of the tomato seedlings. Therefore, it is not recommended for use as a soil amendment in tomato seedbeds.

Our results showed that aromatic plants and their biologically active constituents, when incorporated in soil, fuel the soil food web and boost beneficial nematodes; they are therefore very promising as potential soil amendments. However, before using them as plant or soil biostimulants, herbicides, or pesticides, they need to be tested against several components of the soil environment in order to avoid possible negative effects from their application. Moreover, it is important to consider the decomposition process of botanicals in the amended soil when choosing the right time to sow crops.

## Figures and Tables

**Table 1:** Features of the aromatic plants used as soil amendments. Results of essential oil composition are from Karamanoli et al. [8].

Aromatic Plants	Plant Habit	Essential Oil Yield *	Main Constituents of Essential Oils
*Mentha spicata* L. (spearmint)	Perennial herb	1.31	Carvone (28%) Menthol (23%) Isomenthone (17%)
*Mentha piperita* L. (peppermint)	Perennial herb	0.92	Menthol (40%) Isomenthone (26%) Menthyl acetate (8%)
*Rosmarinus officinalis* L. (rosemary)	Evergreen shrub	2.30	Cineol (45%) Camphor (9%) α-Pinene (12%)

* mL per 100 g d.w. of plant material.

## Data Availability

Data available on request.

## Supplementary Materials

The following supporting information can be downloaded at: https://www.mdpi.com/article/10.3390/life12081121/s1. Table S1: Results of PERMANOVA for the effect of “amendment” and “decomposition” (within factor “amendment”) on the fresh weights of tomato plants. Table S2: Results of PERMANOVA for the effect of “amendment”, “decomposition” (within factor “amendment”), and “tomato presence” (within factor “decomposition” and within factor “amendment”) on soil properties. Table S3: Results of PERMANOVA for the effect of “amendment”, “decomposition” (within factor “amendment”), and “tomato presence” (within factor “decomposition” and within factor “amendment”) on the abundance of the total nematode community and individual trophic groups, on nematode functional indices, and on the nematode metabolic footprint.
