# Supplementary material for: Soil Amendments with Spearmint, Peppermint and Rosemary Enhance the Community of Free-Living Nematodes and Improve Soil Quality, While Having Strikingly Different Effects on Plant Growth
Source: Life (Basel). 2022 Jul 26;12(8):1121. doi: 10.3390/life12081121 (PMC9394304; doi:10.3390/life12081121)
Supplement: Supplementary file 1 [file life-12-01121-s001.zip › life-1791116-supplementary.pdf]

Table S1. Results of PERMANOVA for the effect of "amendment" and "decomposition" (within factor "amendment") on the fresh weight of tomato plants.

**Source**	***df***	***SS***	***MS***	***F***	***p***
Amendment	3	29128.432	9709.477	58.253	0.0002
Decomposition	8	31855.276	3981.909	23.890	0.0002
Residual	24	4000.252	166.677		
Total	35	64983.960			

Table S2. Results of PERMANOVA for the effect of "amendment", "decomposition" (within factor "amendment") and "tomato presence" (within factor "decomposition" within factor "amendment") on soil properties.

	**Source**	*df*	*SS*	*MS*	*F*	*p*
Corg %	Amendment	3	1555.81	518.60	8.43	0.0002
	Decomposition	8	21325.14	2665.64	43.32	0.0002
	Tomato	12	358.02	29.83	0.48	0.9268
	Residual	48	2953.38	61.53		
	Total	71	26192.35			
Norg %	Amendment	3	5084.31	1694.77	237.58	0.0002
	Decomposition	8	454.19	56.77	7.96	0.0002
	Tomato	12	108.85	9.07	1.27	0.2616
	Residual	48	342.41	7.13		
	Total	71	5989.75			
Corg:Norg	Amendment	3	4106.20	1368.73	24.47	0.0002
	Decomposition	8	23734.61	2966.83	53.04	0.0002
	Tomato	12	478.74	39.89	0.71	0.7428
	Residual	48	2685.13	55.94		
	Total	71	31004.68			
NH_4	Amendment	3	41247.38	1349.12	37.51	0.0002
	Decomposition	8	17627.40	2203.43	6.01	0.0002
	Tomato	12	9523.27	793.61	2.16	0.016
	Residual	48	17595.04	366.56		
	Total	71	85993.09			
NO_3	Amendment	3	43853.03	14617.68	128.66	0.0002
	Decomposition	8	17704.76	2213.10	19.48	0.0002
	Tomato	12	1457.22	121.43	1.07	0.3964
	Residual	48	5453.36	113.61		
	Total	71	68468.38			
P	Amendment	3	4849.20	1616.40	5.53	0.0012
	Decomposition	8	23492.36	2936.54	10.05	0.0002
	Tomato	12	12508.23	1042.35	3.57	0.0008
	Residual	48	14020.46	292.09		
	Total	71	54870.26			
K	Amendment	3	55675.08	18558.36	600.64	0.0002
	Decomposition	8	859.56	107.44	3.48	0.0034
	Tomato	12	392.66	32.72	1.06	0.41
	Residual	48	1483.10	30.90		
	Total	71	58410.40			
Mg	Amendment	3	1534.17	511.39	167.88	0.0002
	Decomposition	8	756.10	94.51	31.03	0.0002
	Tomato	12	21.92	1.83	0.60	0.8318
	Residual	48	146.22	3.05		
	Total	71	2458.41			

Table S3. Results of PERMANOVA for the effect of "amendment", "decomposition" (within factor "amendment") and "tomato presence" (within factor "decomposition" within factor "amendment") on the abundance of the total nematode community and individual trophic groups, on nematode functional indices as well as on the nematode metabolic footprint.

	**Source**	***df***	***SS***	***MS***	***F***	***P***
Total abundance	Amendment	3	58425.13	19475.04	65.16	0.0002
	Decomposition	8	52167.13	6520.89	21.82	0.0002
	Tomato	12	4321.70	360.14	1.20	0.1912
	Residual	48	14346.58	298.89		
	Total	71	129260.54			
Abundance of Bacterivores	Amendment	3	54250.55	18083.52	47.1101	0.0002
	Decomposition	8	59870.75	7483.844	19.4965	0.0002
	Tomato	12	4962.521	413.5434	1.0773	0.3814
	Residual	48	18425.11	383.8564		
	Total	71	137508.9			
Abundance of Fungivores	Amendment	3	61103.83	20367.94	43.00	0.0002
	Decomposition	8	31714.70	3964.34	8.37	0.0002
	Tomato	12	5309.89	442.49	0.93	0.5442
	Residual	48	22736.91	473.69		
	Total	71	120865.33			
Abundance of Herbivores	Amendment	3	29851.66	9950.55	4.76	0.0014
	Decomposition	8	47962.77	5995.35	2.87	0.0034
	Tomato	12	19759.88	1646.66	0.79	0.7334
	Residual	48	100237.62	2088.28		
	Total	71	197811.93			
Abundance of Omnivores	Amendment	3	41555.31	13851.77	8.19	0.0002
	Decomposition	8	71737.91	8967.24	5.30	0.0002
	Tomato	12	35112.60	2926.05	1.73	0.027
	Residual	48	81200.45	1691.68		
	Total	71	229606.27			
Metabolic Footprint	Amendment	3	67939.99	22646.66	51.37	0.0002
	Decomposition	8	74237.89	9279.74	21.05	0.0002
	Tomato	12	5886.31	490.53	1.11	0.3346
	Residual	48	21160.06	440.83		
	Total	71	169224.2			
MI	Amendment	3	1587.37	529.12	39.02	0.002
	Decomposition	8	4629.43	578.68	42.67	0.002
	Tomato	12	51.54	4.29	0.32	0.98
	Residual	48	650.95	13.56		
	Total	71	6919.29			
PPI	Amendment	3	31719.50	10573.17	5.76	0.0012
	Decomposition	8	26836.11	3354.51	1.83	0.0888
	Tomato	12	11790.61	982.55	0.53	0.8828
	Residual	48	88169.49	1836.86		
	Total	71	158515.71			
BI	Amendment	3	21652.82	7217.608	25.251	0.0002
	Decomposition	8	62640.49	7830.062	27.3937	0.0002
	Tomato	12	1460.073	121.6728	0.4257	0.9836
	Residual	48	13720.06	285.8346		
	Total	71	99473.45			
EI	Amendment	3	6422.94	2140.98	53.82	0.0002
	Decomposition	8	19308.39	2413.55	60.67	0.0002

	Tomato	12	221.54	18.46	0.46	0.9472
	Residual	48	1909.41	39.78		
	Total	71	27862.28			
CI	Amendment	3	14414.26	4804.75	10.01	0.0002
	Decomposition	8	80976.16	10122.02	21.08	0.0002
	Tomato	12	4089.401	340.78	0.71	0.8034
	Residual	48	23046.88	480.14		
	Total	71	122526.7			
SI	Amendment	3	20978.78	6992.93	4.15	0.0082
	Decomposition	8	34277.32	4284.67	2.54	0.0138
	Tomato	12	30035.50	2502.96	1.49	0.1444
	Residual	48	80828.24	1683.92		
	Total	71	166119.84			
